# A case report of hereditary leiomyomatosis and renal cell carcinoma (HLRCC)

**DOI:** 10.1016/j.eucr.2024.102738

**Published:** 2024-04-10

**Authors:** Sunao Nohara, Shotaro Nakanishi, Tomoki Matsuo, Tomoko Tamaki, Seiici Saito

**Affiliations:** a Department of Urology University of the Ryukyus, Okinawa, 9030215, Japan; b Department of Pathology University of the Ryukyus, Okinawa, 9030215, Japan

**Keywords:** Leiomyomatosis, Renal cell carcinoma, Fumarate hydratase, Cytoreductive surgery, Immune checkpoint inhibitor, Tyrosine kinase inhibitor

## Abstract

Leiomyomatosis and renal cell carcinoma (HLRCC) are rare autosomal dominant cancer syndromes characterized by cutaneous leiomyoma, uterine leiomyoma, and renal cell carcinoma (RCC). RCC in HLRCC is an aggressive metastatic tumor that develops at a young age. Here, we report the case of a patient with HLRCC who was diagnosed after the spontaneous rupture of a renal tumor. The patient underwent cytoreductive surgery, followed by combination therapy with the immune checkpoint inhibitor (ICI) nivolumab and cabozantinib, a tyrosine kinase inhibitor (TKI); however, no improvements were achieved.

## Abbreviations

HLRCC: Hereditary leiomyomatosis and renal cell carcinoma
RCC: renal cell carcinoma
ICI: immune checkpoint inhibitor
TKI: tyrosine kinase inhibitor
FH: fumarate hydratase
CT: computed tomography
MRI: magnetic resonance imaging
PET/CT: positron emission tomography/computed tomography

## 1 Introduction

Leiomyomatosis and renal cell carcinoma (HLRCC) are rare autosomal dominant cancer syndromes characterized by cutaneous and uterine leiomyomas along with renal cell carcinoma (RCC). Germline mutations in the fumarate hydratase (FH) gene cause HLRCC. Moreover, HLRCC is associated with a high risk of developing aggressive and metastatic renal tumors that can occur at a young age. Although surgical therapy is advised for patients without metastasis, the optimal treatment for patients with metastasis has not yet been established. Herein, we report the case of a patient who presented to our hospital with a spontaneously ruptured renal tumor and was later diagnosed with metastatic HLRCC.

## 2 Case presentation

**Fig. 1:**
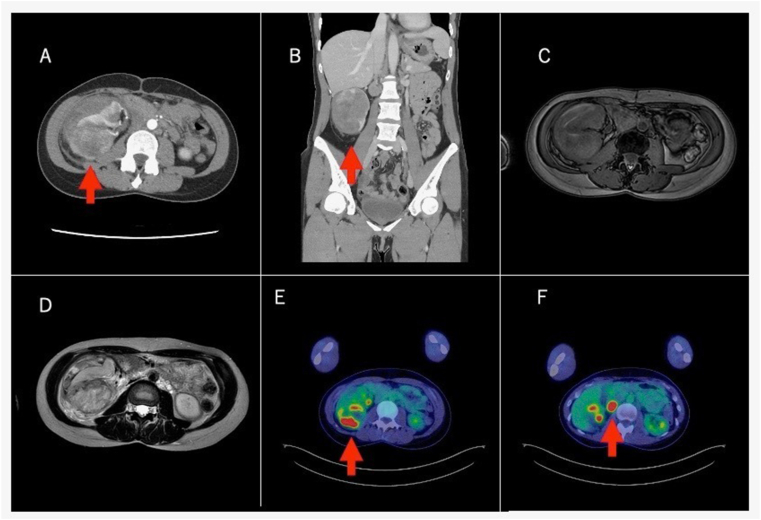
Radiologic findings. Contrast-enhanced computed tomography revealed a 6-cm right renal tumor and retroperitoneal hematoma (A, B). T1-weighted magnetic resonance imaging (C) and T2-weighted magnetic resonance imaging (D) revealing no fat tissue in the tumor. F-18 fluorodeoxyglucose positron emission tomography/computed tomography revealed strong uptake in the right renal tumor with a maximum standardized uptake value of 13.39 (E) and in the hilar lymph node with an uptake value of 15.27 (F).

A 40-year-old woman with a history of multiple uterine leiomyomas underwent laparoscopic hysterectomy and bilateral oophorectomy at 39 years of age. The patient presented to our hospital with a sudden onset of flank pain on the right side. Blood chemistry analysis revealed no evidence of anemia. Contrast-enhanced computed tomography (CT) displayed a 6 cm renal tumor on the right side with progressive contrast, retroperitoneal hematoma due to bleeding from the tumor, enlarged hilar lymph nodes, a right adrenal mass, and multiple small pulmonary nodules (Fig. 1). Contrast-enhanced magnetic resonance imaging confirmed the absence of fat tissue (Fig. 1). Furthermore, 18F-fluorodeoxyglucose–positron emission tomography/computed tomography (PET/CT) revealed uptake of 18F-fluorodeoxyglucose in the right renal tumor, hilar lymph nodes, bilateral pulmonary nodules, right second rib, cervical 4 left transverse processes, thoracic 7 right transverse processes, lumbar 4 vertebrae, and bilateral pelvic bones (Fig. 1).

The patient underwent transcatheter arterial embolization for aggravated right-sided abdominal pain and an enlarged right-sided abdominal mass. Radical right nephrectomy and hilar lymphadenectomy were performed because of right-sided abdominal pain and suspicion of a malignant tumor based on imaging findings.

**Fig. 2:**
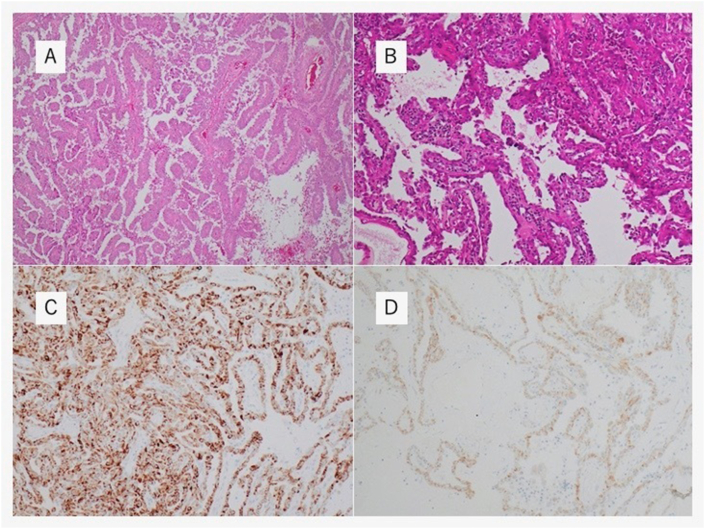
Histopathological findings. (A) H&E staining ( × 40), (B) H&E staining ( × 100): section of the renal tumor exhibited papillary growth pattern. Immunohistochemistry results are positive for alpha–methyl acyl–coenzyme A racemase (C) and cytokeratin 7 (D) H&E, Hematoxylin and eosin.

Pathological examination revealed papillary RCC type 2 with lymph node metastasis (pT3aN2M1). The immunohistochemical results were positive for cytokeratin 7 and alpha–methyl acyl–coenzyme A racemase (Fig. 2). Clinically, HLRCC was suspected because of the presence of cutaneous leiomyoma, leiomyomatosis, and papillary RCC type 2.

Postoperatively, the patient was started on first-line combination therapy with the immune checkpoint inhibitor (ICI) nivolumab and the tyrosine kinase inhibitor (TKI) cabozantinib. Six months after her initial visit, PET/CT imaging revealed no improvement.

## 3 Discussion

HLRCC tends to have an early age of onset, poor prognosis, and aggressive clinical behavior. Patients with HLRCC tend to have uterine and cutaneous leiomyomas, as well as papillary RCC, and approximately 15–30 % of patients with HLRCC develop renal cancer. According to Schmidt et al. ‘s diagnostic criteria for HLRCC, a definitive diagnosis is made by genetic deoxyribonucleic acid testing for germline mutations in the FH gene. A diagnosis of HLRCC is likely when a patient meets the major criteria for the presence of multiple cutaneous leiomyomas with at least one histologically defined lesion. HLRCC was suspected when a patient met at least two of the following minor criteria: 1) solitary cutaneous leiomyoma and family history of HLRCC; 2) type 2 papillary RCC occurring at a young age; and 3) symptomatic multiple uterine leiomyomas.^1^ In which case the patient met the major and three minor criteria and was considered likely to have HLRCC.

In HLRCC, 3%–18 % of patients develop high-grade type 2 papillary RCC, which occurs at a relatively young age and often manifests as lymph node metastasis. The prognosis is extremely poor, with an average survival time of two years after detection. Cases of port-site recurrence have been reported after laparoscopic surgery; radical nephrectomy is recommended for localized HLRCC patients.^2^ Targeted therapies are recommended for metastatic HLRCC. No prognostic data are available regarding cytoreductive surgery in patients with metastatic HLRCC. Iribe et al. reported a case in which a patient with metastatic HLRCC underwent cytoreductive nephrectomy for diagnostic purposes, followed by treatment with nivolumab and axitinib, resulting in long-term survival.^3^ Cytoreductive surgery for metastatic papillary renal cell carcinoma, which is less responsive to TKI compared to clear cell carcinoma, has demonstrated prognostic value in a retrospective study.^4^

To date, no standard therapy has been established for metastatic HLRCC. According to the National Comprehensive Cancer Network guidelines, sunitinib or cabozantinib is recommended as the first-line treatment for non-clear cell RCC. ICI therapy is currently the standard of care for metastatic clear-cell RCC, and recent studies have suggested that ICI therapy is a new option for non-clear-cell RCC. Combination therapy, including MET-specific TKI and cabozantinib, a multi-kinase inhibitor, has presented a relatively good overall response rate. Lee et al. suggested that combination therapy with nivolumab and cabozantinib was effective in patients with papillary RCC.^5^

For this patient, we performed open nephrectomy because of the worsening pain and administered first-line therapy with cabozantinib plus nivolumab, which did not result in an improvement. No genetic test was done because the patient did not request it, and it was not possible to determine the expression of PD-L1 inhibitor, MET or AXL in the tumor.

## 4 Conclusion

Here, we report the case of a patient in whom HLRCC was discovered following rupture of a renal tumor. The patient underwent cytoreductive surgery and ICI plus TKI therapy. Because HLRCC is rare, further case studies are required to establish an appropriate therapy.

## Consent

Written informed consent was obtained for the publication of this case report.

## Funding

This study did not receive any specific grants from funding agencies in the public, commercial, or nonprofit sectors.

## Author contributions

Sunao Nohara and Shotaro Nakanishi: Conceptualization, Investigation, Writing - Original draft preparation, Writing - Reviewing, and Editing. Tomoki Matsuo, Tomoko Tamaki: Writing - Original draft preparation Seiichi Saito: Supervision.

## Section heading

Oncology.

## Declaration of competing interest

The authors declare no conflicts of interest.

## References

[1] Schmidt L.S., Linehan W.M. (2014). Hereditary leiomyomatosis and renal cell carcinoma. Int J Nephrol Renovascular Dis.

[2] Iribe Y., Furuya M., Shibata Y. (2021). Complete response of hereditary leiomyomatosis and renal cell cancer (HLRCC)-associated renal cell carcinoma to nivolumab and ipilimumab combination immunotherapy by: a case report. Fam Cancer.

[3] Yonese I., Ito M., Takemura K., Kamai T., Koga F. (2020). A case of metastatic hereditary leiomyomatosis and renal cell cancer syndrome-associated renal cell carcinoma treated with a sequence of axitinib and nivolumab following cytoreductive nephrectomy. J Kidney Cancer VHL.

[4] Graham J., Wells J.C., Donskov F. (2019). Cytoreductive nephrectomy in metastatic papillary renal cell carcinoma: results from the international metastatic renal cell carcinoma database consortium. Eur Urol Oncol.

[5] Lee C.H., Voss M.H., Carlo M.I. (2022). Phase II trial of cabozantinib plus nivolumab in patients with non—non-clear-cell renal cell carcinoma and genomic correlates. J Clin Oncol.

